# Motor Cortical Activation Assessment in Progressive Multiple Sclerosis Patients Enrolled in Gait Rehabilitation: A Secondary Analysis of the RAGTIME Trial Assisted by Functional Near-Infrared Spectroscopy

**DOI:** 10.3390/diagnostics11061068

**Published:** 2021-06-09

**Authors:** Nicola Lamberti, Fabio Manfredini, Andrea Baroni, Anna Crepaldi, Susanna Lavezzi, Nino Basaglia, Sofia Straudi

**Affiliations:** 1 Department of Neuroscience and Rehabilitation, University of Ferrara, 44121 Ferrara, Italy; fabio.manfredini@unife.it (F.M.); anna.crepaldi@edu.unife.it (A.C.); sofia.straudi@unife.it (S.S.); 2 Unit of Rehabilitation Medicine, University Hospital of Ferrara, 44124 Ferrara, Italy; a.baroni@ospfe.it (A.B.); s.lavezzi@ospfe.it (S.L.); nino.basaglia@unife.it (N.B.)

**Keywords:** multiple sclerosis, rehabilitation, exercise therapy, near-infrared spectroscopy, gait, robot-assisted gait training, biomarker

## Abstract

This study aimed to determine cortical activation responses to two different rehabilitative programs, as measured through functional near-infrared spectroscopy (fNIRS). As a secondary analysis of the RAGTIME trial, we studied 24 patients with progressive multiple sclerosis (MS) and severe disability who were randomized to a regimen of robot-assisted gait training (RAGT) or overground walking (OW). Cortical activation during a treadmill walking task, assessed through fNIRS recordings from the motor and premotor cortexes (M1/PM), was calculated as the area under the curve (AUC) of oxyhemoglobin for each hemisphere and the total area (Tot-Oxy_AUC_). Gait speed, endurance, and balance were also measured, along with five healthy control subjects. At baseline, Tot-Oxy_AUC_ during walking was significantly increased in MS patients compared to healthy people and was significantly higher for those with more severe disabilities; it was also inversely correlated with physical performance. After rehabilitation, significant opposite variations in Tot-Oxy_AUC_ were observed, with activity levels being increased after OW and decreased after RAGT (+242,080 ± 361,902 and −157,031 ± 172,496 arbitrary units, respectively; *p* = 0.002), particularly in patients who were trained at a lower speed. Greater reductions in the cortical activation of the more affected hemisphere were significantly related to improvements in gait speed (*r* = −0.42) and endurance (*r* = −0.44). Cortical activation, assessed through fNIRS, highlighted the brain activity in response to the type and intensity of rehabilitation.

## 1. Introduction

Multiple sclerosis (MS) is a chronic, immune-mediated central nervous system disease with a variable clinical course; this disease can cause dramatic and disabling neurological impairments in young and middle-aged adults [1,2,3].

Walking impairment, which may leave patients increasingly dependent on assistance to walk [4,5,6,7], calls for personalized rehabilitation strategies to improve functional independence and participation in daily activities [1,8,9,10]. Overground walking (OW) is effective for improving mobility in people with MS (PwMS) [11], although it can be associated with a high risk of falls, and patients with severe mobility limitations have a limited ability to take part in OW practice [1,9]. Robot-assisted gait training (RAGT) has been introduced into clinical practice to overcome these limitations and deliver task-oriented, high-intensity gait rehabilitation [12,13]. The efficacy of RAGT among PwMS has been recently reviewed [14,15], and this intervention has been promoted as an option for severely disabled PwMS. Despite the evidence of its clinical efficacy, however, little information is available on the mechanisms underlying the recovery or brain reorganization processes that occur after RAGT. Indeed, especially among diseases where neuroplasticity and adaptive functional reorganization play a role in recovery [16], such as stroke and multiple sclerosis, the ability to measure the effects of the therapies may improve understandings of interventions and recovery mechanisms [16]. Therefore, as a complement to existing validated measures, new assessments, such as the objective quantification of biomarkers of brain activity, may provide additional information to the rehabilitative team [17,18,19].

In particular, the study of cortical hemodynamic activity through the use of functional near-infrared spectroscopy (fNIRS) may offer novel insights regarding rehabilitative interventions [20,21]. This non-invasive technique—which measures cortical oxygenation and, consequently, cortical activation through near-infrared light and does not expose the patient to ionizing radiation—may represent a useful tool in neurorehabilitation [22,23], allowing measurements to be taken during different tasks across various neurological disorders [17]. In PwMS, this promising technique has mainly been exploited using frontal measurements and cross-sectional studies [17,24,25], while any relationships with the outcome measures or the effects of rehabilitation have gone unobserved [17,20,21,22,26]. Increased activation of motor and premotor cortical areas has been found in stroke patients during treadmill walking [27] and in healthy subjects during different walking modalities [28], whereas in PwMS, only the role of the prefrontal cortex during walking has been assessed [17]. No previous studies have explored motor cortical changes after gait rehabilitation, even though it is considered a pivotal factor for gait control. Indeed, motor programs and postural control are generated in the premotor area (PM), whereas the motor command during gait is carried by the corticospinal tract from the primary motor cortex (M1) [29]. The present study, conducted with PwMS who were undergoing gait rehabilitation and were enrolled in the randomized trial RAGTIME [13,30], quantifies and reports the cortical activity recorded through fNIRS during a standardized walking task on a treadmill [31,32,33]. We hypothesized that this novel parameter might represent a marker with features that are useful for the rehabilitation of PwMS. As the purpose of this study, we aim to determine whether (i) different patterns of cortical activity compared to healthy subjects are present in patients with MS; (ii) a relationship with validated, functional measures can be found; and (iii) a selective response to rehabilitation is observable.

## 2. Materials and Methods

### 2.1. Subjects

The patients included in this study sample (*n* = 24) were drawn from participants in the RAGTIME trial. This randomized controlled study compared two different walking rehabilitation protocols in progressive PwMS and severe gait disability (NCT02421731) [30].

Participants were enrolled according to the following inclusion criteria: age between 18 and 65 years and progressive MS with severe gait impairments, defined through an Expanded Disability Status Scale (EDSS) score of 6 to 7. Otherwise, patients were excluded if they met any of the following conditions: inability to perform the timed 25-foot walk test; worsening of MS in the three months before intervention; impaired cognitive function; severe muscle spasticity; clinical conditions in addition to MS that may interfere with the safe completion of the protocol; changes in drug therapy during the study; other rehabilitation treatments; or botulinum toxin injection [30].

Finally, a group of age-matched healthy subjects (*n* = 5), free of medications and known diseases, were included in the study for appropriate comparisons.

The local ethics committee of Ferrara University Hospital approved the present study (number 101/12, on 27 September 2012). The research was carried out according to the Code of Ethics of the World Medical Association (Declaration of Helsinki), and written informed consent was obtained from all participants.

### 2.2. Rehabilitation Treatments

Participants in both rehabilitation groups completed 12 two-hour training sessions over six weeks. The full study protocol has been previously reported [30].

In summary, patients in the RAGT group underwent therapy on a Lokomat treadmill (Hocoma, Volketswil, Switzerland) for approximately 40 min per session. In each training session, the gait speed, body weight support, guidance force, and torque of the knee and hip drives were individually set and progressively adjusted. The patients in the conventional therapy group performed approximately 40 min of physiotherapist-assisted overground walking (OW). Patients were allowed to pause and rest as necessary, and gait speed was set according to their tolerance.

Relative training intensity was calculated as the ratio of the subject’s average speed during the training sessions to the gait speed measured using the timed 25-foot walk test at baseline or the highest speed theoretically attainable by that subject [34]. For the RAGT group, the value obtained was corrected for body weight support (e.g., for 50% of body weight support, the value was multiplied by 0.50). Training intensity was categorized as high or low for each treatment according to a two-quantile distribution, with the median as the cutoff [34].

### 2.3. Outcome Measures

The outcome measures considered in this study were collected at baseline (T0), after six rehabilitation sessions (T1), and at the end of rehabilitation (T2). A single-blind analysis was performed, with outcome assessors and laboratory researchers blinded to group allocation.

#### 2.3.1. Cortical Activation

Changes in brain metabolism during walking were assessed through NIRS using a system (NIRScout, NIRx Medical Technologies LLC, Glen Head, NY, USA) composed of 16 sources and 16 detectors emitting 2 wavelengths of near-infrared light (760 and 850 nm). Hemodynamic signals were recorded at a sampling rate of 3.46 Hz. A standard cap was placed over each participant’s scalp, and sources and detectors were positioned on the measuring cap according to the 10–20 international system. The spatial distribution of the optodes on the cap was chosen to create channels (i.e., source-detector pairs) with standard inter-optode distances of approximately 3 cm. Optodes were placed over both hemispheres, resulting in 48 channels covering the regions of the primary motor and sensorimotor cortices (PMC and SMC).

After the NIRS system was in place, subjects were asked to walk at a speed of 0.2 km/h on a treadmill with a harness to prevent falls (GaitTrainer, Biodex, Shirley, NY, USA). Each 30 s period of walking was followed by 30 s of standing rest, and participants cycled through these 2 phases 4 times, according to the task structure proposed by Kato et al. [27]. For patients with severe ambulatory difficulties, a maximum of 50% body weight support was provided using a harness with a pelvic belt and thigh straps attached to an overhead rigging.

After collection, data were analyzed using NIRSlab software (v. 2017.6, NIRx Medical Technologies LLC, Glen Head, NY, USA). After checking the data quality; removing discontinuities, spike, and movement artifacts; and applying a bandpass filter, we excluded channels with an excessive signal-to-noise ratio or with a specific gain ≥7. Patients who presented more than eight low-quality channels were excluded from the analysis. Then, the optical signals of each channel were converted to oxygenated (oxy-Hb) and deoxygenated hemoglobin concentration changes by using the modified Beer-Lambert law [35].

**Figure 1:**
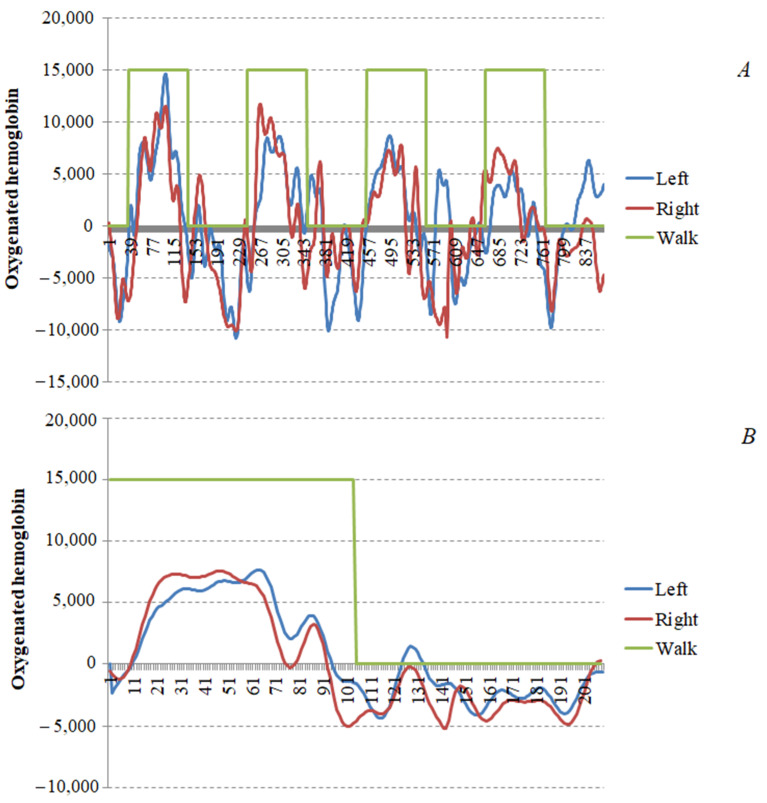
Method of calculation of cortical activation. (**A**): mean track of the 24 channels for each hemisphere during the 4 walking tasks (represented in green); (**B**): mean of the four walking tasks. Cortical activation was calculated as the area under the curve for both hemispheres, as in (**B**).

For quantification of cortical activation (Tot-Oxy_AUC_), we calculated the area under the curve (AUC) of the oxy-Hb trace by summing all the oxy-Hb values during the 30 s walking period for each of the 48 channels. Since the tasks encompassed four 30 s walking bouts, the mean of the four AUCs was calculated. The AUCs of both hemispheres (24 channels each) were also calculated; the hemisphere controlling the more impaired limb was considered the more affected hemisphere (MAff-Oxy_AUC_), and the contralateral hemisphere was considered the less affected of the two (LAff-Oxy_AUC_). In the case of patients with paraparesis, the more impaired limb was identified according to both the physicians’ examination and patients’ perception to determine the more affected hemisphere. An example of AUC quantification is presented in Figure 1.

#### 2.3.2. Performance Parameters

Gait speed, as the primary outcome for the RAGTIME trial [30], was measured by means of the timed 25-foot walk test (T25FW). The patient was instructed to walk 25 ft (7.62 m) as quickly as possible, but safely, using the prescribed assistive devices. The task was immediately administered again by having the patient walk back the same distance. The mean time from the two trials was calculated.

The 6 min walking test was utilized to measure the patient’s capacity for endurance walking [36]. Patients were instructed to walk up and down as far as possible on a 22 m walkway in 6 min without encouragement, and with the option to slow down and rest if necessary. The total distance walked (i.e., 6 min walking distance, 6MWD) was recorded.

Balance was assessed through the Berg Balance Scale (BBS), which measures the patient’s ability to maintain balance statically or while performing functional movements. It includes 14 observable tasks commonly performed in everyday life, with each task measured on a 5-point ordinal scale [37].

### 2.4. Statistical Analysis

The distribution of the data was assessed using the Shapiro-Wilk test. The baseline characteristics of the two rehabilitation groups were compared with an independent samples t-test, a Mann–Whitney U test, or a chi-square test, according to the nature and distribution of the data.

Within-group differences were assessed using a paired samples t-test or Wilcoxon signed-rank test, as appropriate.

Between-group differences in cortical activity were verified through a two-way analysis of variance (factors: treatment, time) or via independent samples *t*-tests or Mann–Whitney U tests to compare between-group differences at the end of treatment, with respect to baseline measurements. In addition, differences in outcome variations according to individual training intensity were assessed through independent samples *t*-tests or Mann–Whitney U tests. Correlations between different outcome measures were assessed using Spearman’s rho.

A *p*-value of 0.05 was considered statistically significant. Data analysis was performed with MedCalc Statistical Software v. 19.8 (MedCalc, Ostend, Belgium).

## 3. Results

Among the 38 patients measured, 24 patients were included in the present analysis, including 12 in the RAGT group and 12 in the OW group. Due to a high number of “low-quality” channels during NIRS measurement, 14 patients were excluded. At baseline, the two groups did not present any differences in clinical features of MS or functional capacity (Table 1).

### 3.1. Cortical Activation at Baseline

At baseline, PwMS exhibited significantly higher cortical activity than healthy subjects, considering both total activation and the activation of the two separate hemispheres (Table 2).

**Figure 2:**
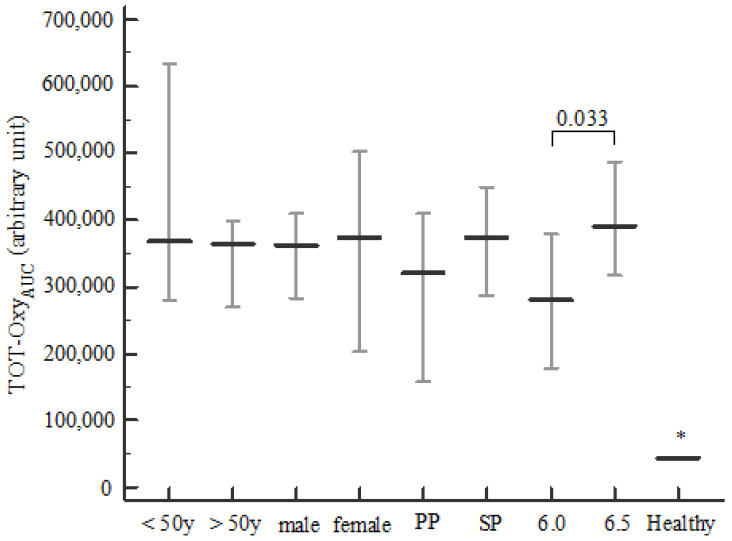
Cortical activation distribution at baseline according to age, sex, phenotype, and EDSS scores. Legend: data are expressed as median values and respective 95% confidence intervals. * *p* < 0.05, in respect to all other parameters.

Cortical activation did not differ by age, sex, or MS phenotype, while significantly higher values were observed for the more disabled patients (EDSS scores of 6.5 vs. 6.0) (Figure 2).

At baseline, the Tot-Oxy_AUC_ values were related only to EDSS scores (*r* = 0.44; *p* = 0.032) when considering clinical parameters, and negatively correlated with performance parameters, including gait speed (*r* = −0.40; *p* = 0.047) and 6MWD (*r* = −0.447; *p* = 0.029).

Interestingly, the MAff-Oxy_AUC_ did not show a significant relationship with any of the clinical or performance parameters. In contrast, the LAff-Oxy_AUC_ was related to EDSS scores (*r* = 0.40; *p* = 0.050) and inversely related to all the performance parameters: gait speed (*r* = −0.54; *p* = 0.007); 6MWD (*r* = −0.56; *p* = 0.043); and BBS (*r* = −0.41; *p* = 0.048).

### 3.2. Cortical Activation Changes after Rehabilitation

Patients in both groups completed all 12 scheduled rehabilitation sessions.

Tot-Oxy_AUC_ showed a significant between-group difference in variation from baseline to the end of treatment, changing in opposite directions between the two groups (RAGT: −157,031 ± 172,496 vs. OW: +242,080 ± 361,902; *p* = 0.002).

A significant group-by-time interaction was also observed for Tot-Oxy_AUC_ (*F* = 5.41 *p* = 0.008), and for MAff-Oxy_AUC_ (*F* = 4.54; *p* = 0.016), between the two groups of patients: these values decreased in the RAGT group and increased in the OW group.

In particular, after RAGT, Tot-Oxy_AUC_ showed a declining trend (*t* = −3.15; *p* = 0.009), with significantly different values observed between T0 and T2 (*p* = 0.028). Decreasing trends were also observed for the two individual hemispheres (MAff-Oxy_AUC_: *t* = −1.87; *p* = 0.08; LAff-Oxy_AUC_: *t* = −1.64; *p* = 0.13).

Patients in the OW group showed the opposite pattern, with the Tot-Oxy_AUC_ significantly increasing (*t* = 2.32; *p* = 0.041), and the individual hemispheres showed similar trends (MAff-Oxy_AUC_: *t* = 1.64; *p* = 0.13; LAff-Oxy_AUC_: *t* = 1.84; *p* = 0.09).

**Figure 3:**
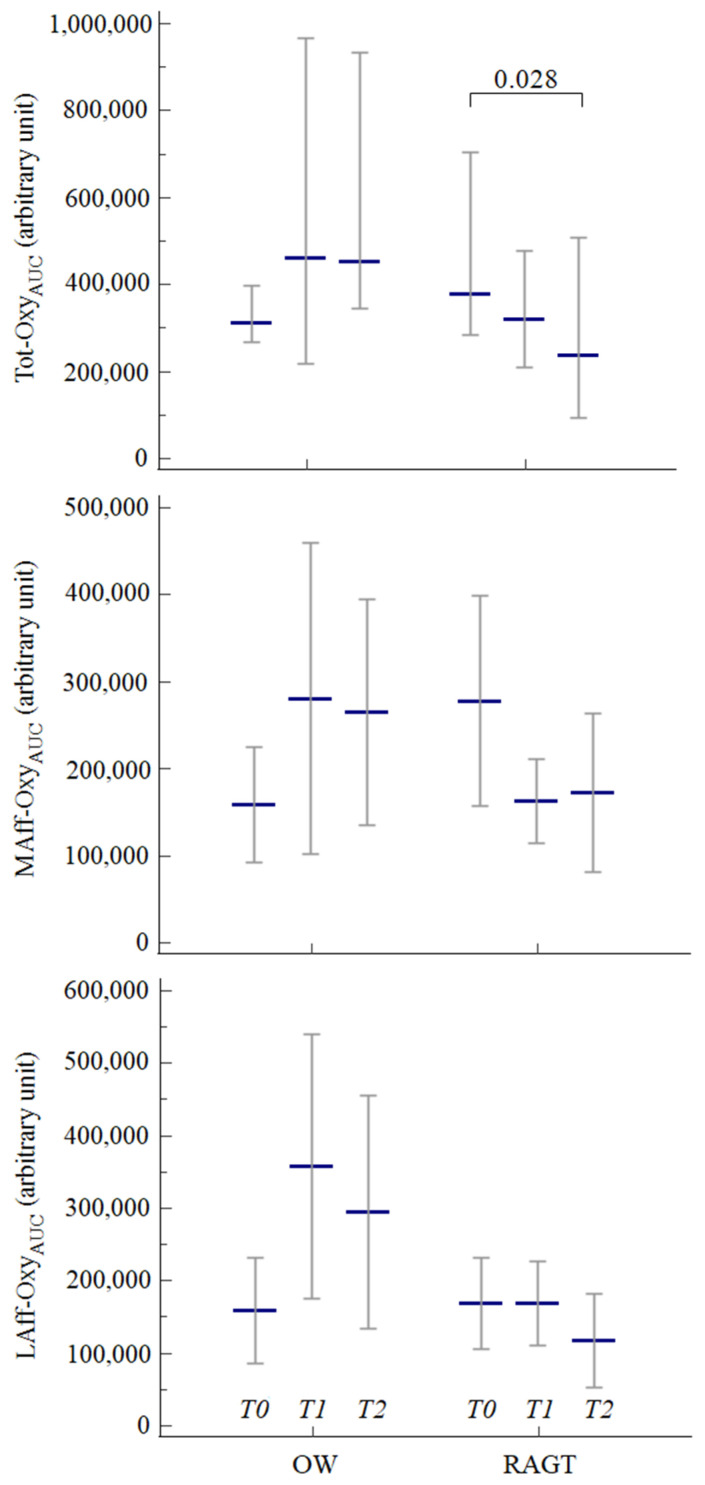
Variations in total and hemispheric cortical activation over time between the two groups. Legend: data are expressed as median values and respective 95% confidence intervals. Abbreviations: OW, overground walking; RAGT, robot-assisted gait training; T0, baseline; T1, after 6 sessions; T2, at the end of treatment.

The data are summarized in Figure 3.

Moreover, among the whole population, a significant direct relation was observed between the RTI and variations in MAff-Oxy_AUC_ (*r* = 0.35; *p* = 0.049) as well as LAff-Oxy_AUC_ (*r* = 0.52; *p* = 0.009).

**Figure 4:**
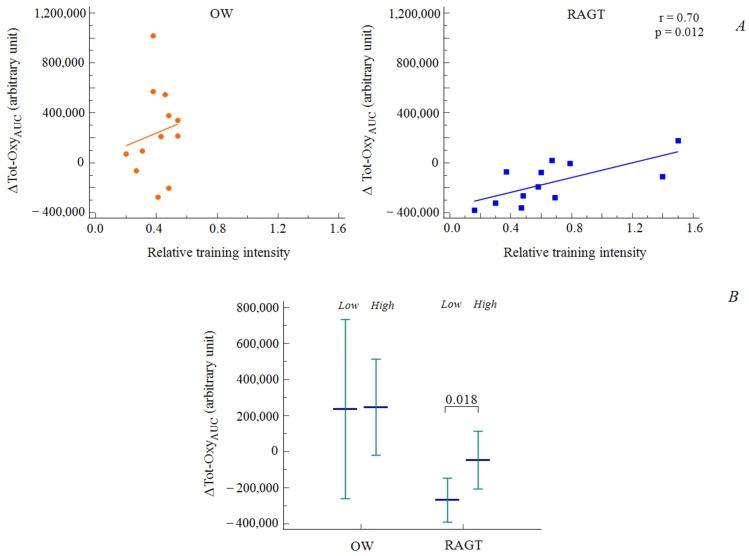
Correlations between variations in cortical activation and relative training intensity (RTI) among the two groups (OW: orange; RAGT: blue) (**A**); variations in cortical activation among the two groups, according to RTI, classified according to the two-quantile distribution (**B**).

When the two treatment groups were analyzed, the RAGT group exhibited significant relationships between the RTI and all three of the cortical parameters (∆Tot-Oxy_AUC_ *r* = 0.70, *p* = 0.012; ∆MAff-Oxy_AUC_ *r* = 0.71, *p* = 0.009; ∆LAff-Oxy_AUC_ *r* = 0.78, *p* = 0.003), whereas no correlations were observed for the OW group (Figure 4). Additionally, in the RAGT group, patients in the lower half of the RTI distribution (RTI < 0.50) showed significantly greater decreases in Tot-Oxy_AUC_ and MAff-Oxy_AUC_ than patients in the upper half. No differences in cortical activation were observed for OW according to the RTI distribution (Figure 4).

### 3.3. Concomitant Variations in Cortical Activation and Functional Parameters

After rehabilitation, all outcomes showed a significant increase, and there were no between-group differences. Specifically, in the whole population, gait speed improved from 0.56 ± 0.32 to 0.61 ± 0.37 ms^−1^ (*p* = 0.013), 6MWD improved from 148 ± 100 to 169 ± 114 m (*p* = 0.009), and BBS improved from 35 ± 13 to 37 ± 13 (*p* = 0.011).

**Figure 5:**
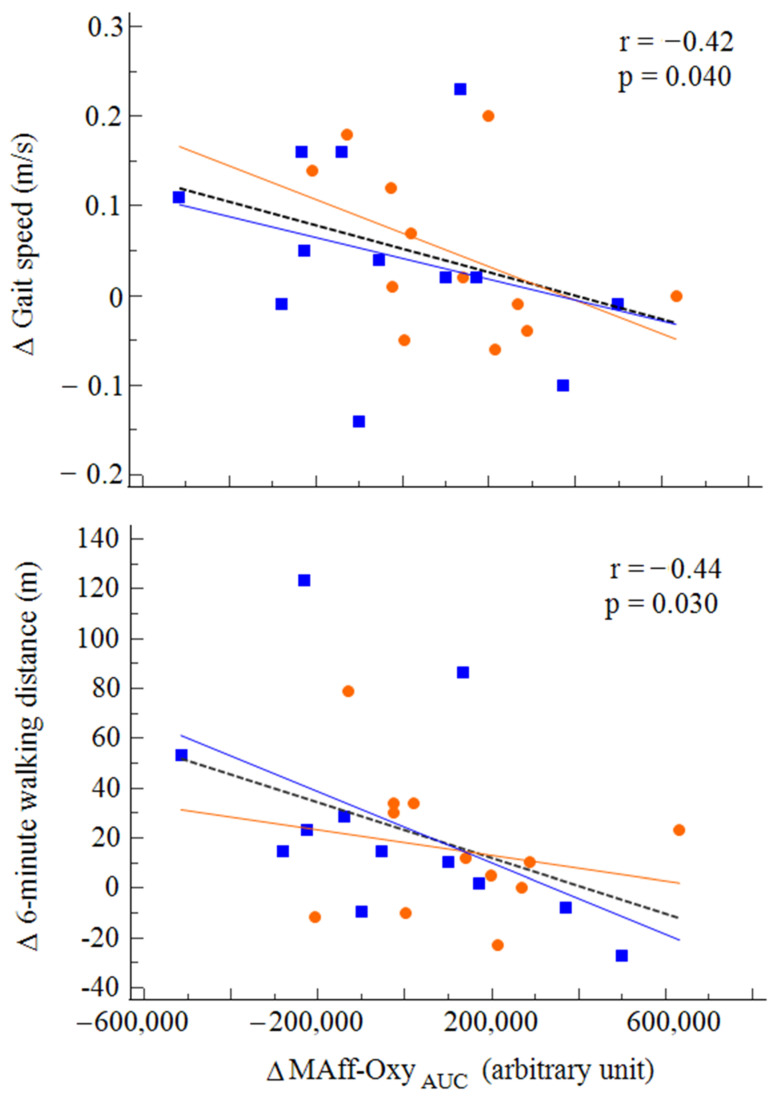
Correlations between variations in cortical activation within the more affected hemisphere and variations in gait speed and walking ability after rehabilitation among the two groups (OW: orange; RAGT: blue).

Moreover, variations in the MAff-Oxy_AUC_ were significantly related to variations in both gait speed (*r* = −0.42; *p* = 0.040) and 6MWD (*r* = −0.44; *p* = 0.030). No significant correlations were observed with variations in Tot-Oxy_AUC_ or LAff-Oxy_AUC_ (Figure 5).

### 3.4. Baseline Cortical Activation and Rehabilitation Outcomes

**Figure 6:**
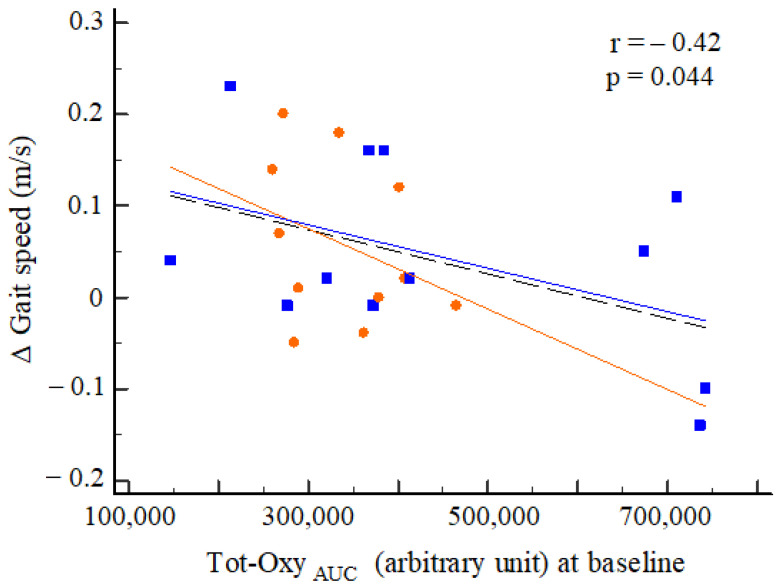
Correlation between cortical activation at baseline and variations in gait speed after rehabilitation among the two groups (OW: orange; RAGT: blue). Legend: tendency lines: orange, OW; blue, RAGT; black dashed, the whole population.

At baseline, Tot-Oxy_AUC_ was significantly correlated with the post-rehabilitation change in performance on the T25FW test (*r* = −0.42; *p* = 0.044), independent of treatment (Figure 6).

No significant correlations were noted for the remaining outcome parameters.

## 4. Discussion

This study is the first to report the quantification of cortical activity through the fNIRS technique, during a walking task—before and after rehabilitation—in severely disabled PwMS. The values of this novel marker in PwMS were significantly higher than those obtained from healthy subjects; additionally, these values were correlated with validated functional outcome measures, including gait speed and endurance, and that were selectively modified by the modality (RAGT or overground) and intensity of rehabilitation. In the absence of a validated protocol for the MS population, the activity in motor and premotor areas was recorded through fNIRS during a treadmill walking task that has been previously used with stroke patients [27].

Additionally, in our study, the analysis in the dynamic phase was based on the changes in oxygenated hemoglobin, a parameter that more accurately reflects cortical activity with a pattern similar to fMRI evaluation [38]. The quantification was carried out through calculating the area under the curve (AUC) in the active phase, as previously performed for the dynamic study of muscle biomarkers [31,32,33].

The baseline comparison with reference values collected from healthy subjects performing the same motor task was the first step in evaluating the reliability of this technological biomarker. In PwMS, the Tot-Oxy_AUC_ was found to be significantly (1.5 to 7 times) higher than that found with healthy subjects, and there was a wide range of inter-individual variability. However, no differences have been found with respect to age, sex, or the MS phenotype (i.e., primary or secondary progressive MS) in our cohort. This reflects how the clinical course of MS and disability progression did not depend on MS phenotype, but instead depended on various factors [39].

Previous studies also observed increased levels of oxygenated hemoglobin and total hemoglobin in the medial primary sensorimotor cortices and the supplementary motor areas during walking [38], and during walking and balance tasks, in older adults—both among patients with Parkinson’s disease [40] and among stroke patients [41]. Among PwMS, increased cortical activity was reported during a dual task that included walking [25,42,43], but the opposite was observed during a finger-tapping motor task [44].

In our study, the degree of variation in Tot-Oxy_AUC_ was not related to the number of steps counted during the task, the individual body weight support provided, or the sex of the patient; however, it was directly related to EDSS scores, although a narrow range of disease severity was present among subjects. An altered level of microvascular saturation (StO_2_) measured in the frontal cortex using a quantitative NIRS-based method was previously reported in patients with MS compared to a control population; the values were correlated with disease duration and EDSS scores [21]. In our study, an interhemispheric asymmetry of activity was also recorded during walking, with higher values in the hemisphere controlling the more affected lower limb than in the contralateral hemisphere. Conversely, in stroke survivors, a population in which an asymmetry of SMC activation was previously observed, a lower activity was recorded for the affected hemisphere [41]. In patients with MS, lateralized brain activity is a consistently reported finding [16] that has been interpreted to be an adaptive mechanism that limits the functional effects of MS damage [45], as impaired interhemispheric inhibition [46,47], or as a possible functional reorganization [16]. In addition, as an aspect of particular relevance, persistent recruitment of the sensorimotor cortex has been linked to poor clinical recovery in this population [48].

The second aspect that should be emphasized is the relationship between the measured cortical activity and the validated functional outcome measures. In our study, a relationship was found, selectively, between total cortical activity—particularly the activation of the less affected hemisphere—and outcome measures reflecting gait, endurance, and balance. This finding was in accordance with previous findings, which highlighted a significant correlation between clinical measures of motor disability and StO_2_ measured in the frontal cortex using quantitative NIRS in PwMS [21]. This significant relationship at baseline showed that higher cortical activity values were consistently associated with worse performance for all parameters. No correlation was observed for the more affected hemisphere, leading us to hypothesize that the hyperactivation of the contralateral hemisphere quantitatively represents a compensatory intervention during walking, which has previously been observed after relapses, in particular [16].

Interesting differential patterns in cortical activation were observed in response to rehabilitation. Tot-Oxy_AUC_ did not initially increase after rehabilitation; however, over time, it exhibited opposite and significantly different trends among the two groups, with activation decreasing in the RAGT group and increasing in the OW group. This finding may appear to be in contrast with other neurological disorders, such as stroke, where a significant increase has been observed in the affected hemisphere after locomotor recovery [41]. Since the majority of MS studies use prefrontal sampling and cognitive or dual tasks [17,42,43], it is difficult to say whether higher or lower cortical activity reflects an adaptive or maladaptive response [16,28]. What is certain, is that apparently healthy subjects present a reduced AUC while walking, and that a reduction in cortical activity closed the gap between MS and healthy subjects on the one hand; while, on the other hand, it was consistently correlated with an improvement in functional capacity, especially among the RAGT group. The reduced cortical activation observed after RAGT may be related to the fact that RAGT requires less energy and imposes less cardiorespiratory stress than OW [49,50], although the partial body weight support provided during the NIRS task may have influenced this issue. Another explanation may be that RAGT, a more controlled, high-dose stepping intervention, is more effective than OW in promoting automatic walking, which decreases motor cortical activation and shifts control from the cortical level to subcortical or spinal levels (central pattern generators).

However, cortical activity is a hemodynamic measure, and pathological hemodynamic features, such as the reported reduction in frontal lobe StO_2_ in MS patients as quantified through fNIRS, may have influenced the data collected [16]. Yang and Dunn hypothesized that StO_2_ may reflect a change in the pattern of supply and demand of oxygen delivery, and indirectly, of the level of inflammation [21]. Differences in StO_2_ were observed in different MS subtypes, as well as reduced blood flow accompanied by local hypoxia [51]; further confirmation was found in the form of high peripheral lactate levels, possibly linked to a systemic hypoxic state [34]. Therefore, the hyperactivation observed in the dynamic state may represent a compensatory response, particularly evident in patients with a more severe disability or a shorter duration of disease. Additionally, mechanisms of hemodynamic compensation could be selectively favored by specific types of rehabilitation and related training factors. To this end, in the present study, we observed that a lower relative intensity was associated with a greater decrease in cortical activation over time, consistent with other peripheral metabolic responses previously observed in a cohort of these patients [34]. The effect of exercise training on cortical plasticity in MS is well-known, but different molecular and instrumental responses have been reported according to training type, volume, and intensity [52,53,54], without the identification of a specific training factor to be addressed.

As a final interesting observation, a higher baseline value of Tot-Oxy_AUC_ was associated with lesser improvements in gait speed and endurance following rehabilitation, regardless of the type of treatment. If corroborated by further studies, this may represent a potentially useful parameter for identifying more responsive patients who might benefit from more frequent rehabilitation treatments.

The present study has some peculiarities compared to previous fNIRS studies [17]; for example, this study featured a longitudinal evaluation, the monitoring of cortical activity outside of the frontal region, and the assessment of possibly related functional parameters and changes in a relatively large and homogeneous population. However, given the novelty of the study, there was no preexisting protocol established for the present purpose; instead, we used a protocol derived from a study of stroke patients with different neurological features and levels of cortical damage. Many points still need to be clarified, even if several similarities have been highlighted.

Moreover, our study has several limitations. First, there was a limited sample size for both PwMS and healthy subjects. Moreover, there were technical limitations, such as the lack of short-channel measurements; we partially resolved this problem by applying a wide bandpass filter [55]. Another limitation is that the choice of a walking task on a treadmill may have favored hyperactivation [56]. Additionally, intersession reliability, previously found to be poor in the prefrontal cortex, was not assessed [24]—as interhemispheric coherence and the separate analysis of the more and less affected hemispheres are questionable in the absence of a unilateral brain lesion, such as an infarct. Finally, in this manuscript, only a whole hemisphere analysis is reported, whereas a focus on smaller areas (such as the PMC or SMC) may have highlighted more precise adaptive responses, as well as the collection of prefrontal cortical activation during walking.

## 5. Conclusions

In conclusion, this study presents a novel parameter of cortical activity, collected through fNIRS during a simple walking task, to assess brain perfusion and recovery through the cortical hemodynamic response to rehabilitation. This novel biomarker differed between healthy individuals and PwMS, with higher values observed in patients with greater disease severity and motor impairment, and it responded differentially according to the types and intensities of rehabilitation. Future studies are needed to confirm the clinical usability of this biomarker in gait rehabilitation studies.

## Figures and Tables

**Table 1:** Patients’ characteristics at the entry.

	RAGT (*n* = 12)	OW (*n* = 12)	*p*
Age, years	56 ± 10	57 ± 11	0.88
Male sex, *n* (%)	5 (42)	8 (67)	0.22
MS duration, years	12 ± 9	20 ± 13	0.12
EDSS	6.3 ± 0.3	6.3 ± 0.3	1
Primary progressive, *n* (%)	6 (50)	4 (33)	0.22
Secondary progressive, *n* (%)	6 (50)	8 (67)	0.22
T25FW speed, m/s	0.50 ± 0.20	0.65 ± 0.40	0.37
6MWT, m	118 ± 55	171 ± 124	0.18
BBS	32 ± 13	36 ± 14	0.43

Abbreviations: MS, multiple sclerosis; EDSS, expanded disability status scale; T25FW, timed 25-foot walk; 6MWT, 6-minute walking test; BBS, Berg Balance Scale.

**Table 2:** Comparison of cortical activation between people with MS and healthy controls.

	PwMS (*n* = 24)	Healthy (*n* = 5)	*p*
Tot-Oxy_AUC_ (a.u.)	382,434 ± 174,813	41,971 ± 8549	<0.001
MAff-Oxy_AUC_ (a.u.)	218,637 ± 162,874	26,428 ± 7488	0.015
LAff-Oxy_AUC_ (a.u.)	163,797 ± 104,461	15,543 ± 2075	0.004

Abbreviations: PwMS, people with multiple sclerosis; Legend: more and less affected hemisphere in healthy subjects, in absence of brain lesions, were calculated according to the dominant limb.

## Data Availability

Research data are available at http://dx.doi.org/10.17632/mv946b4jdp.1.
